# Two Bicycle Dangers

**Published:** 1896-07

**Authors:** 


### TWO BICYCLE DANGERS.

Anything further about the bicycle may seem superfluous. Its hygienic advantages and dangers have been so largely considered the world over that little remains for the present to be said. But with the beginning of a new season it may not be amiss to call attention to two dangers attendant upon the use of the wheel.

Of these, one applies to men chiefly, and consists in the possibility of improperly adjusted saddles, causing an irritation of the perineum. The occasional occurrence of such irritation is unquestionable, and its results concern the posterior urethra rather that the prostrate. Although much is said in speculation of the future "bicycle prostate," that organ is well protected by surrounding tissues, and lies too deep in the pelvis to be seriously influenced by the pressure and resulting congestion from the saddle. It is yet too early to affirm that the increase in bicycle riders will not be accompanied by an increase in the number of hypertrophied prostates; from anatomical grounds, however, such a danger appears slight. It is more probable that any perineal difficulty resulting from bicycle riding will show itself in and around the membranous urethra. It is needless to add that urethræ which have sheltered the gonococcus in their folds are the most liable to become inflamed and the seats of abscesses. Badly fitting saddles have already caused many cases of periurethral abscesses. To avert the extreme pressure on the perineum, and to throw most of the weight on the tubera ischiorum, several "anatomical saddles" have been invented. The principle is the same in all; and although these saddles are less comfortable than the old-fashioned ones, their hygienic superiority is unquestionable. Much the same mechanical advantage may be obtained by tilting forward the ordinary saddle, so that its front part is in a considerably lower plane than the posterior part; the body-weight will then bear chiefly on the ischial tuberosities, and the perineum will touch the saddle but little.

The second danger applies equally to men and women, and has reference to overaction and possible hypertrophy of the heart. If any one stops to count his heart-beats, after even only a moderately brisk spin, it will be found that they are decidedly accelerated, and that the cardiac overaction lasts for a considerable length of time. The amount and duration of the tachycardia will vary, naturally, with the individual, and in the same individual at different times. But the heart should have resumed its normal rate at the end of fifteen or twenty minutes, and if it does not do so it may be inferred that it has more work to do than it can conveniently handle. Accordingly, the bicycle rider, whether man or woman, will only err on the side of safety by noting how much the heart can with safety stand, and will regulate his speed and distance accordingly.—*Am. Med.-Surg. Bulletin.*

